# Action competence in obstetric emergencies—can this be achieved via e-learning? Interprofessional blended learning module on diagnostics and emergency treatment of shoulder dystocia

**DOI:** 10.1186/s12909-023-04335-9

**Published:** 2023-05-18

**Authors:** Verena Stieglitz, Holger Buggenhagen, Kai-Uwe Strelow, Irene Schmidtmann, Christine Skala, Sandra Kurz

**Affiliations:** 1 Rudolf Frey Learning Clinic - Central Educational Platform, University Medical Center of the Johannes Gutenberg University Mainz, Langenbeckstraße 1, 55131 Mainz, Germany; 2 Institute for Medical Biometry, Epidemiology and Informatics (IMBEI), University Medical Center Mainz, Obere Zahlbacher Straße 69, 55131 Mainz, Germany; 3 Clinic and Polyclinic for Obstetrics and Women’s Health, University Medical Center Mainz, Langenbeckstraße 1, 55131 Mainz, Germany

**Keywords:** e-learning, Medical studies, Obstetrics, Shoulder dystocia

## Abstract

**Background:**

Due to the rarity of shoulder dystocia, an obstetric emergency, it is difficult even for experts to develop the appropriate routine in this emergency. Regular further training is therefore recommended for obstetricians and midwives. Evidence is lacking on the extent to which e-learning as a teaching method can be successfully used to acquire these skills and put them into practice. The purpose of this study is to demonstrate how the learning objectives for shoulder dystocia, listed in the National Competence Based Learning Objectives Catalog for Medicine (NKLM, Germany) can be successfully taught in medical studies using a blended learning concept (e-learning and practical application on a birth simulator).

**Methods:**

After completing an e-learning course, final year medical students and midwife trainees demonstrated their action competence for shoulder dystocia procedure on a birth simulator. This transfer of the theoretical knowledge to the case study was assessed using an evaluation form oriented to the recommendations for action.

**Results:**

One hundred sixty medical students and 14 midwifery trainees participated in the study from April to July 2019. Overall, 95.9% of the study participants met the required standards, i.e. achieved very good to adequate performance (Ø good) in simulation training.

**Conclusions:**

E-learning with annotated high-quality learning videos is an excellent way to transfer theoretical knowledge about shoulder dystocia procedures into medical practice on a birth simulator. The learning objectives required by the NKLM for shoulder dystocia can be successfully conveyed to students via the applied blended learning concept.

**Supplementary Information:**

The online version contains supplementary material available at 10.1186/s12909-023-04335-9.

## Background

Prompt action is critical in the presence of shoulder dystocia, an unpredictable and unpreventable obstetric emergency in which the anterior shoulder of the fetus becomes lodged behind the superior symphysis pubis, preventing further delivery [1]. The complications for mother and child are manifold and dependent on the severity of the course [2]. Because of the rarity of shoulder dystocia, which occurs in 0.1% to 2.3% of births, it is challenging even for specialists to have routine knowledge of this emergency presentation [3]. For this reason, regular training based on a fixed flow chart is recommended for obstetricians and midwives [3–6]. A treatment algorithm of manual maneuvers that result in an increase in maternal pelvic diameter, a decrease in foetal shoulder girdle width, or a correction of the foetal birth position is used. This allows the wedged shoulder to be released and the baby to be born [7].

The emergency presentation of shoulder dystocia, is one of the medical conditions listed in the National Competency Based Learning Objectives Catalog of Medicine (NKLM), for which medical students in Germany are expected to acquire competencies [8]. The NKLM, developed by the Society for Medical Education and the Council of Medical Schools in Germany, describes the qualifications of medical school graduates with the aim of supplementing existing lists of medical topics for assessment with learnable competencies [9]. According to NKLM 2.0, students must be able to name prevention options, recognize shoulder dystocia by visual diagnosis, have knowledge of therapeutic options and foetal outcomes, and know that at least a gynecological specialist and a pediatrician must be consulted onset [10]. NKLM states that only knowledge competence is required for the measures of foetal shoulder resolution. However, knowledge is more sustainably anchored by transfer of the theoretically acquired knowledge to practical application on a simulator [11, 12].

Given the many technological advances, a combination of e-learning and face-to-face sessions offers a great way to deliver this knowledge to students quickly and regularly. The term e-learning initially stands for "electronically supported learning" and includes in its definition all forms of learning in which digital media are used for the presentation of learning content or for communication between teacher and learner [13–15]. E-learning offers learners the opportunity to use it independently or within a learning group and to access it at self-determined times. The multimedia presentation of the learning material offers a good form of delivery for different types of learners. Interactive processing elements can enable self-directed, cooperative and individual learning, which promotes autonomy and self-efficacy of the learners [14, 16]. In addition, compared to print media, learning content offered via e-learning can be updated in an uncomplicated and timely manner to ensure that the latest information is always available [14]. With all the possibilities of e-learning, however, it is also important to consider that this teaching method requires a high degree of self-discipline from the students and it is therefore recommended to use platforms that provide feedback on progress to both the students and the teacher. It must be ensured in advance that each student has the technical devices for use and that further learning modules are available for practical application of the theoretically acquired knowledge, so that no social isolation occurs, but group learning processes can take place [17, 18].

Blended learning is a method of learning that combines different learning modalities such as traditional classroom training, e-learning and self-study to create a holistic and sustainable learning experience [19, 20]. At the university, knowledge acquired independently can be applied and consolidated in face-to-face formats, such as simulations or bedside teaching [20].

Within medical studies, e-learning has been identified as a particularly effective learning strategy—along with regular study, good time management, and numerous student-patient contacts [21]. It was shown that when e-learning was used in the teaching of gynecology and obstetrics by medical students, some of them were able to correctly apply the theoretically acquired knowledge in practice after studying the online teaching content only once [22]. Thus, online study outperforms traditional teaching methods, such as frontal lecture, and leads to similar good effects as knowledge acquisition through practical experience [23–25]. However, not all studies can demonstrate the benefits of e-learning. In a meta-analysis, the positive effects of e-learning could be confirmed, but in comparison with non-internet-based teaching methods, such as seminars and hands-on teaching, the effect sizes were only small and rarely statistically significantly increased [26]. It is therefore necessary to use e-learning in a targeted manner.

The extent to which a blended learning concept consisting of e-learning and face-to-face teaching can be used promisingly for the students' competence development was investigated. To this end, a new teaching concept was developed for the interdisciplinary obstetrics course of the Department of Gynecology and Obstetrics at University Medical Center Mainz, Germany. As a case study, a blended learning course was designed for the obstetric emergency of shoulder dystocia. Medicine students and midwifery trainees were assessed for their level of competence on shoulder dystocia after completing an approximately one-hour e-learning course [27]. The goal was evidence-based use of novel teaching methods, in this case explicitly e-learning in the context of blended learning concepts. In addition, the authors wanted to find a way to teach action competence on this rare obstetric emergency of shoulder dystocia already in medical school, before the knowledge must be practically applied on the real patient in an emergency without hesitation.

## Methods

### Hypothesis and research questions

The null hypothesis, which had to be disproved, states that e-learning is not suitable as a teaching method so that medical students can competently apply the theoretically learned knowledge independently in the obstetric course to the obstetric emergency of shoulder dystocia on the simulation trainer.

The research questions attempt to clarify the extent to which e-learning can be successfully used as a teaching method in medical studies to acquire skills in the diagnosis and treatment of shoulder dystocia and put them into practice. The focus here is on the learning objectives for shoulder dystocia listed in the NKLM and the extent to which these can be successfully taught using a blended learning concept.

### Research design and study population

The quantitative, monocentric and prospective study took place from April to July 2019 as part of the compulsory internship in obstetrics at University Medical Center Mainz, which every final-year student must complete. The study population included all final year students (*n* = 160) as well as final year trainees (*n* = 14) of the affiliated school of midwifery. The distribution to the small groups of the different internship days was done in a randomized way. The performance of the participants in applying the knowledge learned in the e-learning on the birth simulator was collected by the teachers in a pseudonymized way.

### Blended learning scheme

**Fig. 1:**
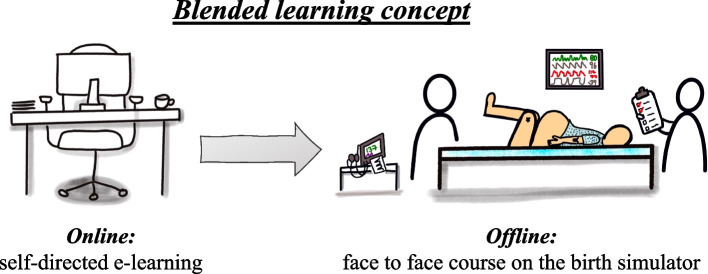
“Blended learning in obstetrics” near here. Caption: Structure of the blended learning concept for teaching the treatment of shoulder dystocia

Blended learning formats combine online learning and face-to-face classroom sessions with the aim of creating a more sustainable learning experience by linking theoretical knowledge with practical application. As an online learning element of the birth course, participants were provided in advance with approximately one hour of e-learning containing theoretical knowledge about the diagnosis and treatment of shoulder dystocia. In the face-to-face format, the previously learned knowledge was applied in a simulation of shoulder dystocia on a birth simulator. This blended learning format is visualized in Fig. 1.

### Design of the e-learning

**Fig. 2:**
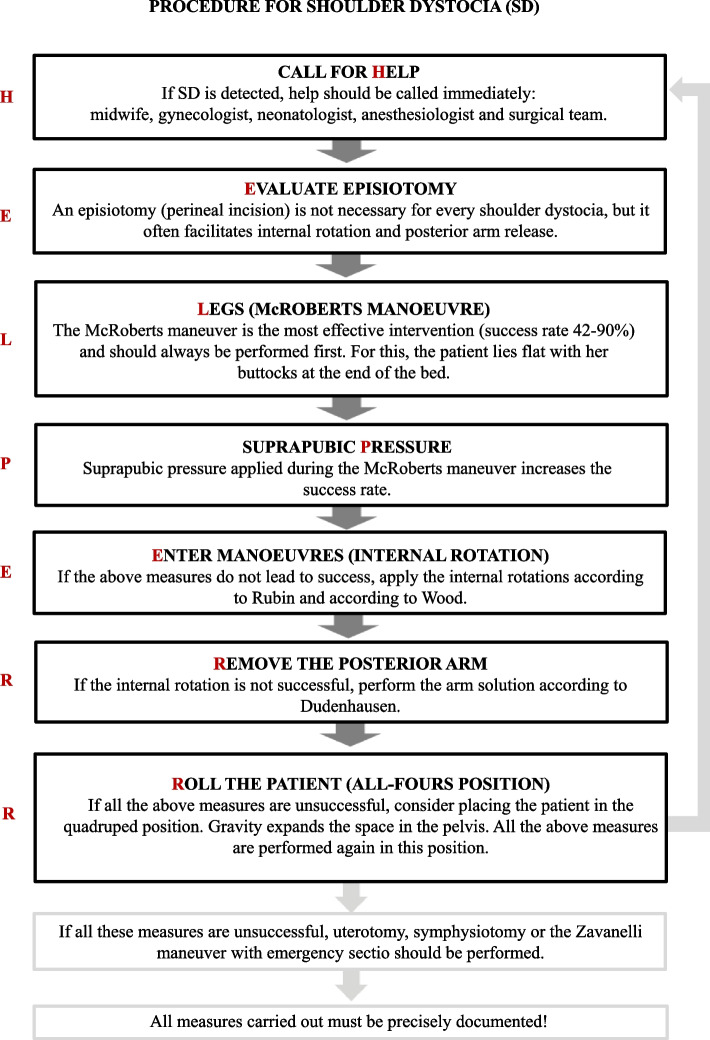
“Procedure for Shoulder Dystocia” near here. Caption: This procedure for shoulder dystocia was provided to the study participants in e-learning [7, 34]

**Fig. 3:**
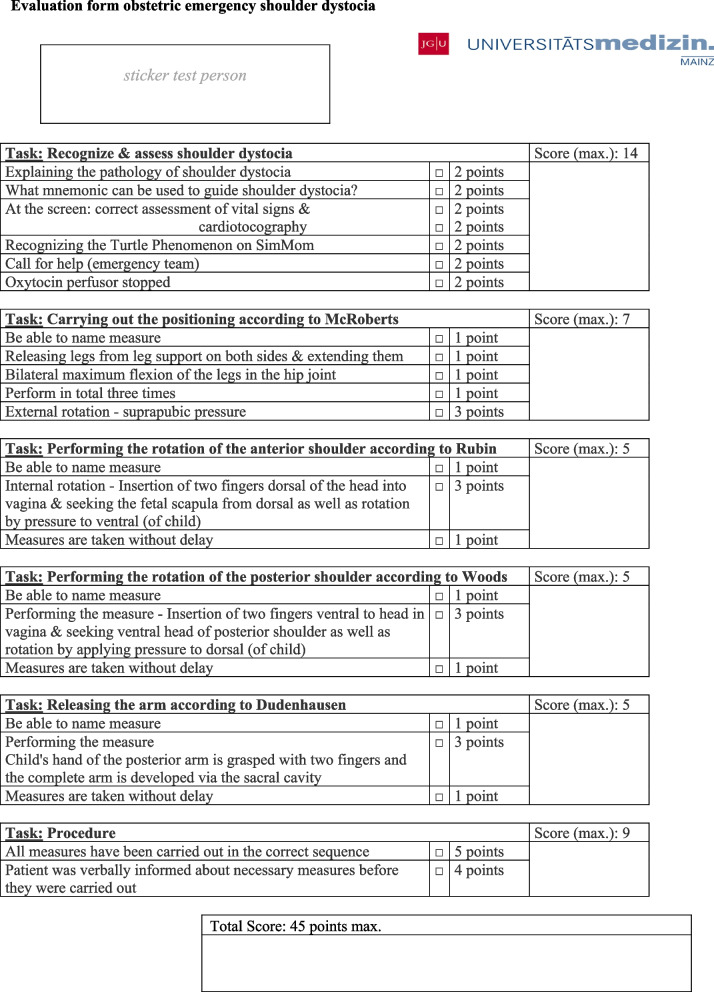
“Evaluation form” near here. Caption: Evaluation form for assessing study participants' performance in simulation training (based on recommended actions [32])

For creation of the e-learning course within the blended learning module, the open source management system "OpenOLAT" was used, which offers the possibility to import, create and visualize own learning content as well as to analyze its usage [28]. "OpenOLAT"-based e-learning had already been used in previous sections of the medical studies in Mainz, so students were already familiar with the application. A comprehensible use could also be ensured for university transfer students, since only login data was required for the application and the system offers a support area [29]. The learning objectives were collected with the help of the Learning Opportunities, Objectives and Outcomes Platform "LOOOP", a web portal which is used internationally for the competence-oriented conception, development, evaluation and accreditation of several study programs at various medical faculties, and is being further developed by the Charité, University Medical Center Berlin, Germany [30]. In this way, students were able to prepare themselves independently based on the learning objectives provided in the e-learning. The learning content of shoulder dystocia was conveyed in the course using five different videos and accompanying texts on definition, diagnosis and manual measures according to their namesakes McRoberts, Rubin, Woods and Dudenhausen (cf. Figs 2 and 3). For this purpose, students and midwifery trainees were directed to a 3D-animated training program of the German Society for Prenatal and Obstetric Medicine, in which the maneuvers were explained in detail [31]. The content of the e-learning was based on recommended actions of the Advanced Life Support in Obstetrics (ALSO®, [32]). The ALSO program was developed by the American Academy of Family Physicians (AAFP), one of the largest medical organizations in the United States [33]. This course trains physicians, midwives, nurses, and other health care providers involved in potential emergencies in maternal and infant perinatal care, with the goal of reducing morbidity and mortality in both groups [33]. The procedures of the course were used as a guide for conducting the blended learning concept in the present study. The treatment algorithm used, called the "HELPERR" scheme [34, 35] is a mnemonic, that offers a structured framework to the maternity team for releasing the foetal shoulder during shoulder dystocia [7]. Figure 2 shows the "HELPERR" scheme. After completion of the e-learning course, study participants were offered a self-assessment quiz.

### Sequence of the childbirth course

All participants were provided with the e-learning module in advance. The students had no contact with the topic of shoulder dystocia in their previous medical studies; the trainees had discussed this topic theoretically in advance but had not yet applied it practically either. With the acquired background knowledge, the study participants were assessed for their level of competence based on the performance recorded in the simulation training.

The study participants went through the simulation training individually using a standardized flow chart. The scenario started with a theoretical unit in which the background knowledge of the pathology of shoulder dystocia and the mnemonic "HELPERR" was tested. After that, the condition of the woman in labor and the fetus was assessed by means of vital signs and cardiotocography. The shoulder dystocia was presented with the delivery simulator SimMom® from the company Laerdal. The first task was to recognize the prolonged course of birth based on the turtle phenomenon, a clinical sign in which the foetal head retracts back onto the maternal perineum after delivery [34]. Secondly, based on the first letter of the "HELPERR" scheme, they had to request help immediately. In the further course, it was necessary to correct the attitude anomaly promptly. The study participants had to name the measure and explain how they would also educate about it in the delivery room and perform it on the simulator. In the order of the measures, points were awarded for correct prioritization according to the "HELPERR" scheme. After this instruction the attending instructor assisted in performing the McRoberts maneuver which, as a non-invasive measure, often already leads to release of the shoulder by extension and flexion of the birthing woman's legs [36]. Subsequently, after unsuccessful performance of the McRoberts maneuver three times in combination with suprapubic pressure, an intravaginal maneuver had to be applied. The fetus was set in the shoulder straight position several times, so that the study participants had to perform internal rotation according to Rubin and Woods as well as Dudenhausen arm release (cf. Fig. 2), all of which are more invasive measures to be used only when the previous ones have not been successful [37]. In the case of the final maneuver, the case study dictated that, if performed correctly, the shoulder would release, and the study participants would be able to fully deliver the child.

The local ethics committee of the Medical Association of Rhineland-Palatinate, Germany, confirmed that there were no risks or burdens for the participants. Nevertheless, each simulation was discussed with the participants to offer an opportunity to express psychological stresses and concerns that may arise in connection with the emergency obstetric situation.

### Learning objectives of the evaluation form

The evaluation form (see Fig. 3) is divided into three sections based on the learning objectives of the NKLM 2.0 (see Background) and international guidelines of shoulder dystocia: (I.) Recognizing and assessing shoulder dystocia, (II.) Performing the necessary measures and (III.) Procedure [10, 38–40]. The first section (I.) focuses on explaining the pathology of shoulder dystocia and recognizing it based on the turtle phenomenon and being able to assess the condition of mother and child based on vital signs and cardiotocography, as well as immediately alerting the emergency team. Within the second section (II.) the individual measures of releasing shoulder dystocia are evaluated. Here, the students perform the leg positioning according to McRoberts, in which the fetal shoulder is to be released under the symphysis by bending the legs in the hip joint, just as in the internal maneuvers according to Rubin and Woods. The last measure tested is the arm releasing according to Dudenhausen, in which the posterior arm is developed. In the third part (III.) it was important to carry out all mentioned measures in the right order and to inform the woman in labor about them.

### Collection and evaluation of the study data

The evaluation form oriented to the ALSO® recommendations for action [32] and the learning objectives of NKLM 2.0 [10] served as the basis for assessing the performance achieved in the simulation training (see Fig. 3). Due to the rarity of shoulder dystocia, there is a lack of international recommendations with a high level of evidence. However, review of shoulder dystocia guidelines from various countries (e.g. USA, UK, Australia, Germany [41–44]) as well as numerous reviews [34, 38–40] revealed a consensus in the recommended interventions, which is applied in the ALSO® courses. The consensus from the international guidelines and reviews was used to create the evaluation form of the present study.

The participants' action competence was measured by means of a score formed of a total score of the individual items. In this process, the weighting of the items was determined in a review process. The applicability of the assessment sheet was evaluated before the start of the study in two trial runs with five study participants each.

The statistical analysis of the scores in the evaluation forms was carried out with IBM® SPSS® Statistics 23, graphical representations with Microsoft® Excel®, under the supervision of the Institute for Medical Biometry, Epidemiology and Informatics (IMBEI), University Medical Center Mainz. Descriptive measures for the quantitative characteristics were mean, median, upper quartile, lower quartile, and outliers, and boxplots were created to represent the characteristics of the participant group. Absolute and relative frequencies were determined for categorical characteristics and presented in the bar charts. A comparison of the study participants' action competencies was made between training groups, previous experience, previous professional training and gender, in each case using the Mann–Whitney-U-test performed at the significance level of α = 5% = 0.05.

## Results

All 10th semester medicine students at the Johannes Gutenberg University in Mainz, Germany (*n* = 160, 93 female, 67 male) and all 3rd year students of the School of Midwifery of the University Medical Center Mainz (*n* = 14), participated in the study. The male students were on average 27 years old and the female students 23 years old.

Before starting medical school, 79 study participants (49%) had already completed a vocational education. The three most frequently mentioned professional trainings included paramedic or emergency medical technician (45%, *n* = 36), nurses (25%, *n* = 20), and physiotherapist (11%, *n* = 9). Fifty (31%) of the study participants indicated that they had previous experience in dealing with obstetric emergencies during their previous work as paramedics or during their internships in medical school.

The evaluation showed that every participant had dealt with the e-learning in advance, at least in part. 95 participants stated that they had completed the e-learning in full. 12 participants stated that they had worked through 90% of the e-learning, 31 participants 80%, five participants 70%, 15 participants 60%, four participants 50%, three participants 40%, two participants 30% and six participants 20%. Accordingly, none of the participants appeared in the birth course without preparation.

**Fig. 4:**
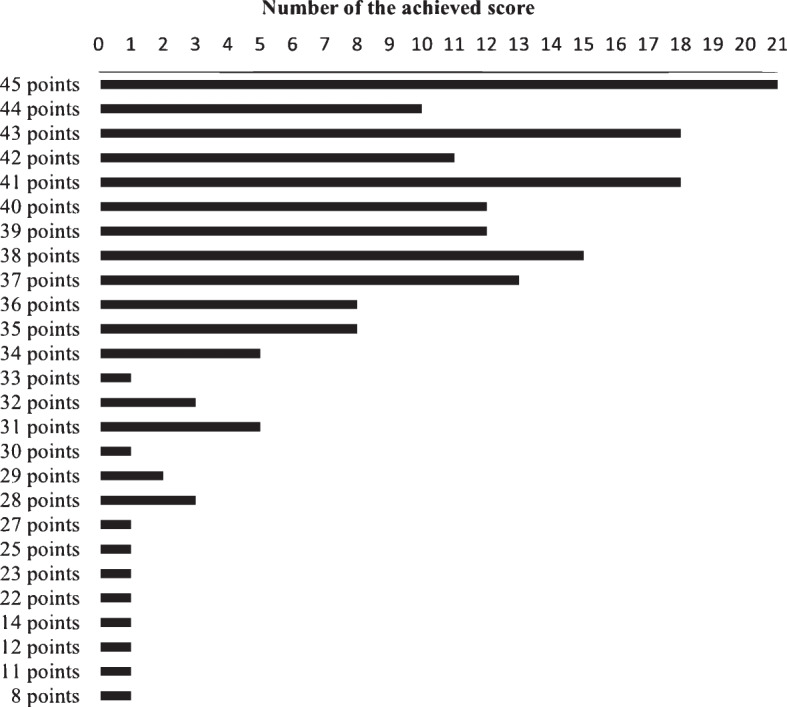
“Score of study participants in simulation training” near here. Caption: Total score achieved by study participants in simulation training (Max: 45 points, Min: 0 points, Passing limit: 28 points)

To evaluate the transfer of knowledge acquired in the e-learning to practice, the performance collected via the evaluation form (cf. Fig. 3) was assessed. A maximum of 45 points could be achieved. Sixty study participants scored more than 90%, 70 study participants scored between 80 and 90%, 25 study participants scored between 70 and 80%, 12 scored between 60 and 70%, and 7 scored less than 60% (see Fig. 4). Overall, 96% of the study participants had performed in accordance with the requirements (60% pass rate). The mean score of the performances achieved was 38.5 points.

**Fig. 5:**
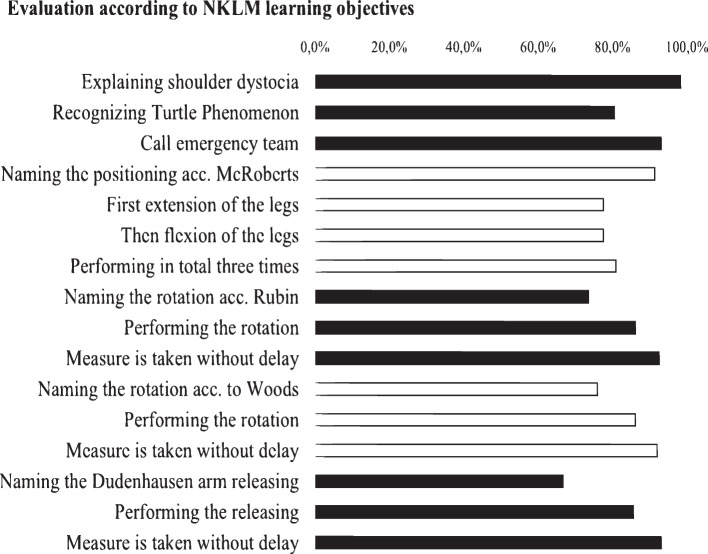
“Meeting the requirements of NKLM” near here. Caption: Evaluation of performance of the study participants according to the learning objectives required by the NKLM for shoulder dystocia [10]

Figure 5 shows the study participants' level of competencies within the learning objectives established by the NKLM. Over 98% of study participants were able to explain the entity of shoulder dystocia and its preventive measures. About 93% immediately informed the emergency team after realizing the urgency. The learning objective "recognize the turtle phenomenon" was achieved by 81% of the study participants. In the sub-area of positioning according to McRoberts, more than 90% were able to name the measure, but the correct execution of this proved to be difficult (only 78%—81% achieved the maximum score, depending on the sub-item). In the case of the rotation according to Rubin and Woods as well as the arm release according to Dudenhausen, the weak point was in remembering the names of the measures. More than 85% of the study participants were able to perform the above-mentioned measures correctly, and more than 92% did so within a period appropriate to the critical situation. Study participants who were unable to achieve the learning objectives stated that they had not sufficiently engaged with the e-learning due to personal motivational fault or technical problems.

A comparison of medical students (*n* = 160) and midwifery trainees (*n* = 14) was performed to analyze the collected performances against the background of biographical data. Medical students scored a mean of 38.3 points (standard deviation (SD) 6.5) on simulation training, whereas trainees scored 40.6 points (SD 3.3). There was no significant difference between medical students and trainees: *p* = 0.204 (α = 0.05).

In addition, the extent to which a vocational education completed prior to medical school had an impact on performance was examined. The medical students who had completed a vocational education beforehand scored a mean of 38.9 points (SD 5.86), while the others scored 38.2 points (SD 6.71). Again, there was no significant group difference: *p* = 0.602 (α = 0.05).

Furthermore, the influence of previous experience in dealing with obstetric emergencies among medical students was evaluated. The medical students who had experienced obstetric emergencies beforehand scored a mean of 38.5 points (SD 5.78), whereas the students with no previous experience in this field scored 38.2 points (SD 6.82). Similarly, there was no significant group difference: *p* = 0.687 (α = 0.05).

When evaluating a possible influence of the study participants' gender, male participants scored a mean of 38 points (SD 6.9), while females scored 38.8 points (SD 5.9). Again, no significant group difference was found: *p* = 0.683 (α = 0.05).

**Fig. 6:**
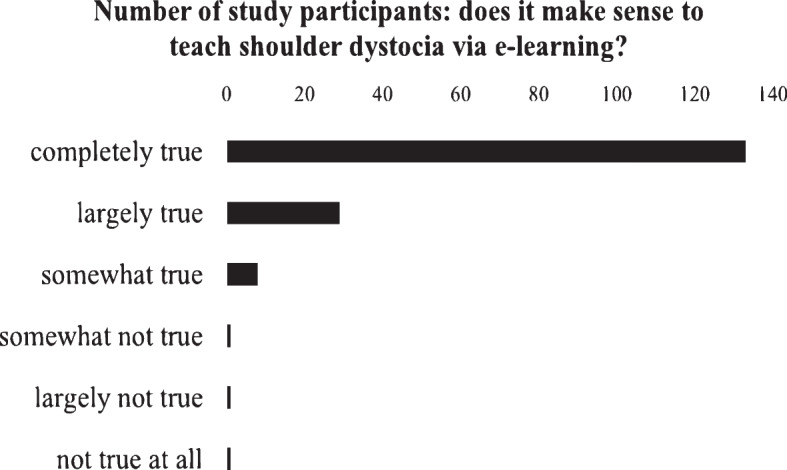
“Supporting of e-learning as a learning method” near here. Caption: Study participants' assessment of the extent to which shoulder dystocia should be incorporated into the obstetrics curriculum via e-learning

Of the study participants, 94% reported that they enjoyed learning during simulation training. When asked to assign a school grade to the "HELPERR" module established for the study, 114 study participants assigned the grade "very good," 50 assigned the grade "good," 7 assigned the grade "satisfactory," while the grades "sufficient," "poor," and "insufficient" were assigned once each. When asked whether study participants thought it was useful for shoulder dystocia to be taught in this way in obstetric education, 133 responded "strongly agree," 29 "mostly agree," 8 "somewhat agree," and 4 disagreed (see Fig. 6).

## Discussion

Analyzing the use of online learning is an important aspect of tracking student progress and thus identifying any early dropouts or weaker students and providing targeted support [16]. Fifty-seven percent of participants had completed all the e-learning in advance, and another 38% had completed more than half. This supports the assumption that the skills were acquired by using the e-learning provided. For the participants who had performed in the lower range, it was possible to demonstrate that they had not used the e-learning to its full extent. The fact that almost 96% of the study participants had achieved the required learning objectives, and that an average of more than 80% of the total score could be achieved, shows that e-learning can be a promising teaching tool for obstetric emergencies. It seems to enable students and trainees to correctly apply the acquired knowledge in practice without significant prior practical experience.

The evaluation of the surveyed performance (cf. Fig. 5) showed that the learning objectives established by the NKLM could be achieved. In principle, theoretical background knowledge can be conveyed well via e-learning. Ninety-three percent of the study participants, remembering the "HELPERR" scheme, had alerted help immediately after recognizing the shoulder dystocia, which is an important aspect in the professional handling of emergency situations [35]. Another relevant aspect, the correct performance of measures to release shoulder dystocia, was mastered by at least 77% of the study participants (depending on the measure). The transfer of theoretical knowledge into practical application is generally considered more demanding than the mere recall of theoretical expertise. At this point, e-learning alone would reach its limits, which is why the practical application of measures to release shoulder dystocia is enormously important. According to the NKLM, only knowledge competence is required for the measures to release the foetal shoulder, but this knowledge remains more sustainable if the students also have action competence [10–12]. It was striking that only about 78% of the study participants were able to perform the positioning according to McRoberts without error. Some participants reported that the information in the e-learning was not sufficiently comprehensible. In comparison, the rotations according to Rubin and Woods as well as the arm release according to Dudenhausen, which are among the more complex and invasive measures in the context of handling a shoulder dystocia, could be put into practice comparatively well by the study participants using the e-learning module. The use of the mnemonic "HELPERR" had supported the study participants in performing the measures in the correct sequence.

Neither a previous vocational education, gender nor former professional experience had a significant influence on the results among the students. The midwifery students, at the end of their obstetrics-oriented training, were not significantly superior to the students who had no contact with obstetrics until the 10th semester. Consequently, appropriately designed e-learning can facilitate good performance in practical application, regardless of prior experience. Based on the evaluation forms, it can be said that the e-learning teaching method is an effective way of imparting knowledge of the learning objectives on shoulder dystocia established by the NKLM. To maximize the successful transfer of the knowledge acquired in e-learning about the recognition and treatment of shoulder dystocia into practical application on the simulator, it is important to provide a manageable, easy-to-use e-learning offer and to enable the prompt practical application of the knowledge in small groups with feedback from the instructor.

The personnel and time requirements as well as the costs incurred for the creation of e-learning modules must be considered. When creating the teaching content, it is also important not only to carefully examine the professional content, but also to select the pedagogical aspects of the teaching, so that high-quality knowledge can also be conveyed in an appealing and sustainable manner [17]. Methodological aspects that positively influence participants' autonomous use can include the quantity on the one hand, as well as the importance of completed learning activities and activities that explicitly require presence on the platform on the other [45]. The creators of the e-learning researched in this study had deliberately limited the amount of knowledge to be imparted to about one hour of learning time and communicated to students in advance the obligation to complete the e-learning. Furthermore, especially with action-based e-learning, the practical application of knowledge learned online strengthens participant engagement, which reinforces the use of a blended learning format for teaching action skills [45, 46]. The saving of personnel and spatial resources in the clinical everyday life and the option of asynchronous teaching makes it possible to call up the contents at any time. This is also reasonable for the high acceptance of this teaching by the students. The modules created also offer an opportunity to prepare the young doctors in further training for their first delivery room assignment. Thus, the theoretical basics are available on demand for everyone working in obstetrics. This teaching method provides a safe way for teaching the care of rare clinical cases. The present study demonstrated that knowledge can be successfully taught and retrieved via blended learning. In follow-up studies, the sustainability of blended learning formats must be examined, for example, in the application of the acquired competencies in residency training. In the case of digital teaching formats, it is also important to check that all students have the technical means to use them, and that smooth retrieval of the learning content is guaranteed at the same time [17]. Furthermore, a format should always be chosen that is simply structured and appealing, so that independent use is encouraged [17].

## Conclusions

The results show that it is possible to teach competencies in diagnostics and treatment of the obstetric emergency shoulder dystocia by means of e-learning in such a way that they can be applied in practical simulation without the possibility of prior practice and independently of other prior experience. In this way, not only can the learning objectives required by the NKLM for shoulder dystocia be successfully conveyed by means of e-learning, but more advanced action competencies can also be acquired. Blended learning should be increasingly used not only in student teaching, but also in residency training, to safely manage emergencies that rarely occur in clinical practice.

## Practice points

- E-learning is suitable for teaching diagnostics and treatment as well as the learning objectives required by the NKLM for the releasing of shoulder dystocia.
- Medical students can convert the knowledge competence acquired via e-learning into action competence in the practical simulation, independent of prior experience.
- E-learning should be applied in the context of blended learning concepts to consolidate knowledge.

## Supplementary Information

**Additional file 1.** 

## Data Availability

All data generated or analysed during this study are included in this published article [and its supplementary information files].

## Abbreviations

ALSO: Advanced Life Support in Obstetrics
HELPERR: Help, Episiotomy, Legs, Pressure, Enter, Remove, Roll
LOOOP: Learning Opportunities, Objectives and Outcomes Platform
NKLM: National Competence Based Learning Objectives Catalog for Medicine
